# Development and Application of a Multiplex Real-Time Fluorescent PCR Assay for the Detection of Common *Lactobacillus* Species in Food

**DOI:** 10.3390/molecules31111790

**Published:** 2026-05-23

**Authors:** Qin-Feng Qu, Qing-Ping Zhang, Yi Yu

**Affiliations:** Key Laboratory of Milk and Dairy Products Detection and Monitoring Technology, State Administration for Market Regulation, Shanghai Institute of Quality Inspection and Technical Research Co., Ltd., Shanghai 200233, China; zhangqp@sqi.org.cn (Q.-P.Z.); yuyi@sqi.org.cn (Y.Y.)

**Keywords:** multiplex real-time fluorescent PCR, *Lactobacillus*, *L. acidophilus*, *L. plantarum*, *L. rhamnosus*, *L. paracasei*, multiplex identification

## Abstract

*Lactobacillus* species are widely used in various food products, including conventional food products, dairy products, and health food products. To achieve the desired functional properties, manufacturers commonly incorporate two or more distinct *Lactobacillus* species during production. In this study, a multiplex PCR detection method was developed for four *Lactobacillus* species commonly used in food based on TaqMan real-time fluorescent PCR technology, enabling the efficient and rapid identification of multiple *Lactobacillus* strains in food matrices. The research team selected and validated four representative species—*Lactobacillus rhamnosus*, *Lactobacillus plantarum*, *Lactobacillus acidophilus*, and *Lactobacillus paracasei*—as targets for the multiplex PCR assay, designing specific primer–probe combinations for each. The accuracy and reliability of the detection method were rigorously evaluated through a series of validation experiments, including the assessment of primer–probe specificity, optimization of fluorescent signal labeling chemistries, determination of the limits of detection for individual strains, evaluation of the method’s repeatability, and analysis of commercial food samples. The results demonstrated that the selected primer–probe sets exhibited no cross-reactivity in the multiplex system and specifically amplified their target *Lactobacillus* species, with no amplification observed for non-target strains. The established method achieved a minimum LOD for *L. acidophilus* of 10^2^ CFU/g and showed high repeatability across replicates. Furthermore, the successful detection of labeled *Lactobacillus* strains in commercial products confirmed the method’s practical applicability. Therefore, the developed multiplex real-time PCR assay provides a reliable, sensitive, and high-throughput tool for the simultaneous detection of multiple *Lactobacillus* species in complex food products and holds potential for application in quality control, product authentication, and regulatory compliance monitoring.

## 1. Introduction

### Research Background and Significance

Probiotics are defined as live microorganisms that, when administered in adequate amounts, confer a health benefit on the host via colonization of the human body and modulation of the microbiota composition in specific host niches. These beneficial microbes promote host health by regulating mucosal and systemic immune functions, maintaining intestinal microbiota homeostasis, enhancing nutrient absorption, and preserving intestinal integrity. Probiotics comprise either single microbial strains or well-characterized, defined microbial mixtures that exert validated health-promoting effects [1,2,3]. Among diverse probiotic lineages, lactic acid bacteria (LAB) represent the most extensively investigated and widely recognized group, garnering substantial research attention globally [4].

Lactic acid bacteria are a taxonomically diverse group of microorganisms characterized by their ability to produce high concentrations of lactic acid through carbohydrate fermentation [5]. With a long-standing history of safe utilization and broad natural distribution, LAB exhibit remarkable species diversity. To date, approximately 40 genera and over 300 species of LAB have been identified and documented in natural ecosystems [6,7]. In the food industry, LAB have emerged as highly sought-after functional ingredients in recent years, with widespread applications in conventional foods, dairy products, health food products, and special dietary formulations. Within the LAB group, the genus *Lactobacillus* serves as a pivotal functional strain in food fermentation, playing a critical role in determining final product quality and food safety [8].

LAB can confer distinct health-promoting and functional properties upon food matrices [9,10,11]. Through the metabolic production of organic acids, bacteriocins, hydrogen peroxide, diacetyl, and other bioactive metabolites, LAB mediate a range of beneficial physiological functions, including the regulation of gastrointestinal microbiota balance, alleviation of chronic metabolic disorders, modulation of host immune responses, and improved nutrient absorption, via synergistic interactions between the bacterial cells and their metabolites. Additionally, these metabolites effectively inhibit the proliferation of food spoilage microorganisms, thereby extending the shelf life of fermented food products [12].

*Lactobacillus* species identification has traditionally relied on phenotypic and biochemical characterization, including morphological observation and physiological profiling. In recent years, however, advanced molecular biological techniques—such as polymerase chain reaction (PCR) and 16S rDNA sequencing—have been developed and widely adopted for accurate *Lactobacillus* species identification [13,14,15]. Numerous novel detection and strain typing methodologies have emerged. For instance, researchers have completed whole-genome resequencing of 185 *Streptococcus thermophilus* strains isolated from natural fermentation systems using the Illumina Hiseq high-throughput sequencing platform [16]. Zhang et al. [17] established a T-RFLP platform for *Lactobacillus* detection in microecological environments using species-specific primers targeting the 16S-23S rRNA gene intergenic spacer region; Chen et al. [18] employed the SMM system to screen for specific gene sequences of *Lactobacillus plantarum*. Furthermore, pulsed-field gel electrophoresis (PFGE) [19], restriction fragment length polymorphism (RFLP) analysis [20], metagenomic sequencing, average nucleotide identity (ANI) calculation, and MALDI-TOF-MS protein fingerprinting [21,22] have been gradually implemented for probiotic identification and detection assay development.

Nevertheless, notable limitations persist in current *Lactobacillus* identification technologies. First, the majority of existing detection methods are restricted to the identification of a single *Lactobacillus* species [23,24], lacking comprehensive and systematic approaches for the multiplex identification of multiple target species. Second, several reported multiplex PCR identification methods focus on strains not listed in the List of Strains Permitted for Use in Food Products issued by the National Health Commission of the People’s Republic of China [25], which restricts their practical applicability. Third, certain advanced identification techniques entail high detection costs and stringent technical requirements for operating personnel, hindering their widespread implementation in routine laboratory testing.

Regarding the three limitations above, this study aimed to establish a multiplex real-time fluorescence PCR assay for the multiplex identification and detection of *Lactobacillus* species that are listed in the List of Strains Permitted for Use in Food Products [25]. Among them, the *Lactobacillus* species *L. acidophilus*, *L. plantarum*, *L. rhamnosus*, *L. paracasei*, *L. delbrueckii* subsp. *Bulgaricus*, *L. casei*, and *L. reuteri* are widely used in the food industry, so this study focused on these seven. By optimizing a multiplex primer pool and refining the multiplex amplification reaction system, this study sought to develop a high-throughput detection methodology, providing a rapid, efficient, accurate, and cost-effective technical tool for routine *Lactobacillus* testing in food matrices [26,27,28].

## 2. Results

### 2.1. Screening Primer–Probe Combinations for Multiplex Real-Time Fluorescence PCR System [29]

#### 2.1.1. Screening of Modification Groups for Multiplex Real-Time Fluorescence PCR

**Figure 1:**
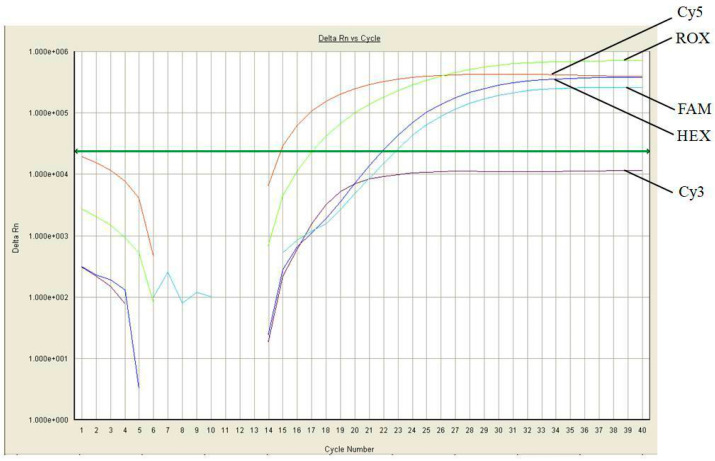
The fluorescence signal intensities of Cy5, ROX, FAM, HEX, and Cy3. (The horizontal green line represents the threshold of fluorescence signal intensity.).

**Figure 2:**
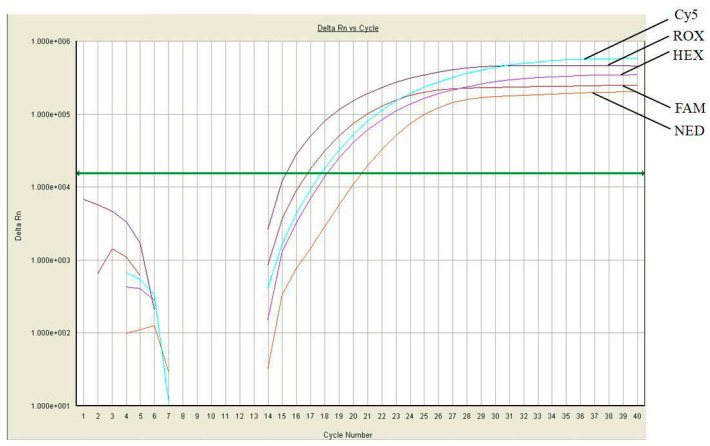
The fluorescence signal intensities of Cy5, ROX, FAM, HEX, and NED. (The horizontal green line represents the threshold of fluorescence signal intensity.).

After a literature review and repeated comparisons, and considering the performance of the equipment used, FAM, Cy3, Cy5, HEX, and ROX were initially selected as the modification groups for the multiplex fluorescence PCR. Based on the experimental results for the 5′ end modification groups obtained from the five fluorescence channels, it was found that the fluorescence signal intensity of the Cy3 modification group was relatively weak, being almost two orders of magnitude lower than the fluorescence signal intensities of the other four modification groups (Figure 1). This showed that Cy3 could not meet the requirements for multiplex real-time fluorescence PCR experiments in this reaction system. The 5′ end modification group was replaced with NED, which has the same emission band as Cy3, to resynthesize the probe. The fluorescence signal of this probe was as strong as the signals of the other four modification groups and could well meet the fluorescence signal requirements for analyzing multiple target species in the same reaction system (Figure 2). Therefore, FAM, NED, Cy5, HEX, and ROX were finally selected as the modification groups for the multiplex fluorescence PCR.

#### 2.1.2. Specificity Verification of Multiplex Real-Time Fluorescence PCR Primer–Probe Combination

The primer–probe sets for the seven target *Lactobacillus* species, including *L. acidophilus*, *L. plantarum*, *L. rhamnosus*, *L. paracasei*, *L. delbrueckii* subsp. *bulgaricus*, *L. casei*, and *L. reuteri* were combined for multiplex real-time fluorescence PCRs to individually detect the seven target *Lactobacillus* species. If the Ct value ≤ 30.0, the sample was determined to be positive; if the Ct value > 35.0, the sample was determined to be negative. If 30.0 < Ct value ≤ 35.0, the amplification was repeated once. If the Ct value was still ≤ 35.0 after the second amplification, the sample was determined to be positive; if the Ct value was> 35.0 after the second amplification, the sample was determined to be negative.

It was found that, when the primer–probe set for *L. delbrueckii* subsp. *bulgaricus* was combined with the sets for *L. casei* or *L. paracasei* in the same reaction system, the DNA of *L. delbrueckii* subsp. *bulgaricus* was specifically amplified by the primer–probe set for *L. casei* or *L. paracasei*, indicating that *L. delbrueckii* subsp. *bulgaricus* showed cross-reactivity with respect to the target sites of *L. casei* or *L. paracasei* in the multiplex fluorescence PCR system, and the primer–probe set for the two target sites could not be used in the same reaction system (Table 1, Table 2 and Table 3). Additionally, when the primer–probe set for *L. reuteri* was in the same reaction system as the set for *L. casei* or *L. paracasei*, the DNA of *L. reuteri* was specifically amplified by the primer–probe set for *L. casei* or *L. paracasei*, indicating that *L. reuteri* was cross-reactive with respect to the target sites of *L. casei* or *L. paracasei*, and the primer–probe sets for the two target sites could not be used in the same reaction system (Table 4 and Table 5).

Following the combinatorial optimization of various target gene loci, four qualified multiplex fluorescence PCR primer–probe systems were ultimately screened and validated for further testing. The four optimized combinations were as follows:

1. *L. rhamnosus* + *L. plantarum* + *L. acidophilus* + *L. delbrueckii* subsp. *bulgaricus*;
2. *L. rhamnosus* + *L. plantarum* + *L. acidophilus* + *L. reuteri*;
3. *L. rhamnosus* + *L. plantarum* + *L. acidophilus* + *L. casei*;
4. *L. rhamnosus* + *L. plantarum* + *L. acidophilus* + *L. paracasei*.

All four multiplex real-time fluorescence PCR systems exhibited minimal to no cross-interference during the amplification process, and all the target bacterial strains were efficiently and specifically amplified. Notably, no amplification signals were detected for non-target bacterial strains, and any observed non-specific amplifications yielded Ct values greater than 35.0, indicating negligible non-specific binding (Table 6, Table 7, Table 8 and Table 9).

Theoretically, numerous combinatorial schemes are feasible for multiplex fluorescence PCR assays targeting the seven selected *Lactobacillus* species. However, experimental validation revealed significant mutual interference between the primer–probe sets for certain target strains when co-existing in a single reaction system, leading to severe non-specific amplification. Specifically, consistent non-specific amplification of *L. delbrueckii* subsp. *bulgaricus* or *L. reuteri* strains was observed when the primer–probe combination set for *L. delbrueckii* subsp. *bulgaricus* or *L. reuteri* and *Lactobacillus casei* or *Lactobacillus paracasei* were in the same reaction. Accordingly, the maximum number of target strains that could be reliably detected simultaneously in this multiplex real-time PCR system was limited to four.

**Figure 3:**
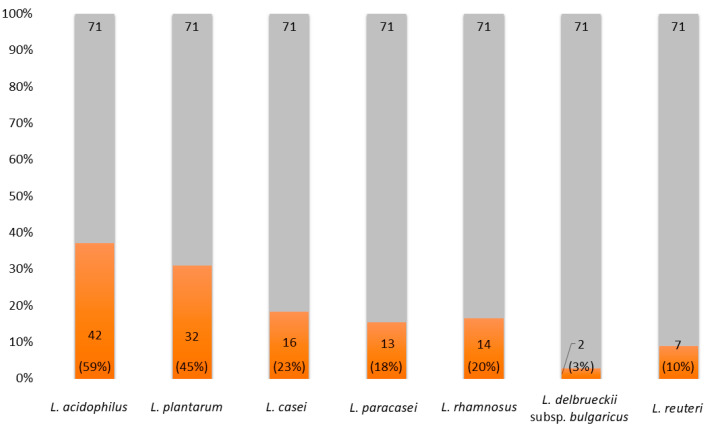
Proportions of *Lactobacillus* in 71 batches of products (gray: total number of food samples; orange: number of strains used in food samples).

Based on this market survey and practical application demand, flexible primer–probe combinations can be adopted for multiplex real-time fluorescence PCR detection according to the specific strain profiles labeled for commercial probiotic products. This study surveyed the strain composition and application of *Lactobacillus* in commercial probiotic products, analyzing a total of 71 batches of food samples encompassing health food products, solid beverages, pet foods, and snack foods. Among these products, the majority incorporated two or more of the seven *Lactobacillus* species investigated in this research (Figure 3). Ultimately, the primer–probe sets for *L. rhamnosus*, *L. plantarum*, *L. acidophilus*, and *L. paracasei* were selected as the final optimized target combination for validating the established multiplex PCR method, owing to their high prevalence in commercial products and excellent amplification compatibility in the reaction system.

### 2.2. Specificity Verification of Multiplex Fluorescence PCR Primer–Probe

As shown in Table 10, the multiplex primer–probe sets demonstrated clear specific amplification for the target *Lactobacillus* species, with Ct values ranging from 15.15 to 23.68. No significant amplification was observed for non-target species, as all the corresponding Ct values exceeded 35.0. These results confirm the high specificity of the selected multiplex primer–probe combinations.

### 2.3. Limit of Detection (LOD)

Multiplex real-time fluorescence PCR amplification was performed using target *Lactobacillus* bacterial suspensions. As detailed in Table 11, the limits of detection (LODs) for the four target species were determined as follows: *L. paracasei* at 10^5^ CFU/g, *L. plantarum* at 10^4^ CFU/g, *L. rhamnosus* at 10^3^ CFU/g, and *L. acidophilus* at 10^2^ CFU/g. Notably, the optimized multiplex PCR method established in this study enables the reliable detection of *L. acidophilus* at a minimum concentration of 10^2^ CFU/g, demonstrating favorable sensitivity for practical food sample testing.

We found that the LOD values differ substantially across species, from 10^2^ CFU/g to 10^5^ CFU/g. This may be attributed to two possible reasons. First, the differences in LOD among species may be attributed to interspecific variations in DNA extraction efficiency. Different *Lactobacillus* species possess unique cell wall structures and compositions, which influence the efficiency of cell lysis by the extraction buffer and subsequently lead to differences in DNA recovery rates. Second, the inherent differences in amplification efficiency among the primer–probe sets designed for each species may also contribute to the variation in detection limits across species.

### 2.4. Repeatability Validation of the Multiplex PCR Assay

To verify the accuracy and reproducibility of the established method, mixed bacterial suspensions containing *L. acidophilus*, *L. plantarum*, *L. rhamnosus*, and *L. paracasei* were prepared for repeatability testing. Six independent replicate experiments were performed in parallel using the optimized multiplex real-time fluorescence PCR primer–probe set, in accordance with standardized laboratory protocols, to assess assay repeatability. As presented in Table 12, all four target Lactobacillus species were consistently and accurately detected in the mixed bacterial suspensions across all six replicate tests; the Ct values were 17.02 ± 0.35, 17.02 ± 0.77, 20.08 ± 1.10, and 21.14 ± 0.47. These results confirm that the developed multiplex real-time fluorescence PCR method exhibits excellent intra-assay repeatability and reliable detection performance for the simultaneous identification of the four target strains.

### 2.5. Validation Using Commercial Food Samples

Ten commercial food samples were collected for practical applicability testing, consisting of five solid beverage products and five health food products, all of which were labeled to contain two or more of the target strains: *L. rhamnosus*, *L. plantarum*, *L. acidophilus*, and *L. paracasei*. All samples were subjected to detection analysis using the optimized multiplex primer–probe set developed in this study (Table 13). The results demonstrated that the established multiplex real-time fluorescence PCR assay specifically and accurately identified the declared target *Lactobacillus* strains in all the tested commercial food samples, with no non-specific amplification or false-positive signals observed. Collectively, these findings verify that the developed detection method possesses strong practical applicability and reliable accuracy for the simultaneous identification of the four target *Lactobacillus* species in commercial food samples.

## 3. Discussion

This study established a multiplex identification and detection method for *Lactobacillus* species based on multiplex real-time fluorescence PCR. By leveraging the high efficiency and cost-effectiveness of this technique, which enables the simultaneous amplification of multiple target DNA fragments in a single reaction, the developed assay allows for the simultaneous detection of four *Lactobacillus* species. This method focuses on the seven *Lactobacillus* species commonly used in the food industry, covering the main strains used in commercial food production. The method was validated through assessments of primer–probe specificity, the assay’s repeatability, the detection sensitivity for each target bacterium, and the analysis of commercially available samples.

We were surprised to observe non-specific amplification when the primer–probe set for *L. delbrueckii* subsp. *bulgaricus* was combined with the sets for *L. casei* or *L. paracasei* in the same reaction system. A similar phenomenon occurred between the primer–probe set for *L. reuteri* and those for *L. casei* or *L. paracasei*. It is hypothesized that these results may be attributed to partial sequence matching with non-target templates. According to the experimental verification, four distinct multiplex primer–probe sets are suitable for simultaneous detection: 1. *L*. *rhamnosus* + *L*. *plantarum* + *L*. *acidophilus* + *L*. *delbrueckii* subsp. *Bulgaricus*. 2. *L*. *rhamnosus* + *L*. *plantarum* + *L*. *acidophilus* + *L*. *reuteri*. 3. *L. rhamnosus* + *L*. *plantarum* + *L. acidophilus* + *L. casei*. 4. *L. rhamnosus* + *L. plantarum* + *L. acidophilus* + *L. paracasei*. We chose the fourth multiplex primer–probe set for method validation based on the prevalence of corresponding *Lactobacillus* species in commercial products.

From the results for detection sensitivity, we can see that the limits of detection (LODs) for the established method were as follows: *Lactobacillus paracasei*, 10^5^ CFU/g; *Lactobacillus plantarum*, 10^4^ CFU/g; *Lactobacillus rhamnosus*, 10^3^ CFU/g; and Lactobacillus acidophilus, 10^2^ CFU/g. Compared with the reported LODs for an existing published method [30], there are some differences. The observed discrepancies may be attributed to the fact that the LODs for this published method were derived from a singleplex fluorescent PCR assay, whereas our study designed a multiplex primer–probe system, in which competition or interference among different primer–probe sets may occur. Additionally, factors such as the concentrations of primers and probes, and the amount of DNA template added, could also influence the experimental results. Therefore, to achieve stable and reliable LODs, it is strongly recommended that comparisons and validations be conducted under consistent experimental conditions.

In the repeatability validation of the multiplex PCR assay, the RSD of target *Lactobacillus* strains ranged from 2.07% to 5.84%, showing that we can identify them reliably. Finally, when we used the sample brought from the market for practical applicability testing, we could identify the strains well. The results confirmed that, without the need for prior bacterial culture, all four target Lactobacillus species could be effectively detected, provided that their concentrations in the sample met the respective detection sensitivity thresholds. Using this method, we can reliably and effectively identify multiple target Lactobacillus strains used in this experiment in products.

## 4. Materials and Methods

### 4.1. Strains and Samples

#### 4.1.1. Stains

*L. acidophilus* (CICC6075), *L. plantarum* (ATCC 8014), *L. rhamnosus* (CICC6162), *L. paracasei* (CICC 6107), *L. delbrueckii* subsp. *bulgaricus* (CICC 6097), *L. casei* (CICC 6117), *L. reuteri* (CICC 6118), and *B. animalis* (ATCC 27673) were incubated in MRS broth medium in an anaerobic atmosphere at 37 °C. *S. thermophilus* (CICC 6038) and *L. lactis* (ATCC 11454) were incubated in MRS broth medium in an aerobic atmosphere at 37 °C.

#### 4.1.2. Commercial Samples

We used five solid beverages and five health food products, each containing two or more of the *L. rhamnosus*, *L. plantarum*, *L. acidophilus*, and *L. paracasei* strains.

### 4.2. Instruments, Equipment, and Reagents

#### 4.2.1. Instruments and Equipment

qPCR instrument (ABI 7500Fast) (Applied Biosystems (Thermo Fisher Scientific), Foster City, CA, USA); micro-volume UV analyzer (DeNovix DS-11) (DeNovix Inc., Wilmington, DE, USA); vortex oscillator (Votex 2) (Scientific Industries (SI). New York, USA); centrifuge (Eppendorf Centrifuge 5415R) (Eppendorf SE, Hamburg, Germany); micro-pipettes; constant-temperature water bath; high-pressure sterilizer.

#### 4.2.2. Reagents

MRS broth medium; CTAB lysis solution (2% cetyltrimethylammonium bromide, 1.4 mol/L NaCl, 0.1 mol/L Tris-HCl, 0.02 mol/L ethylenediaminetetraacetic acid (EDTA) pH 8.0); Proteinase K (≥600 mAnson U/mL); lysozyme solution (20 mg/mL); 1 × TE buffer (0.01 mol/L Tris-HCl, 0.002 mol/L mM EDTA pH 8.0); real-time fluorescence PCR premix (2 × Premix Ex Taq™ probe); Tris phenol:chloroform:isoamyl alcohol (25:24:1); chloroform:isoamyl alcohol (24:1); ethanol.

#### 4.2.3. Primers and Probes

The primers and probes [24,31] used in this study are those employed in established single-plex PCR methods, which have been officially published in authoritative Chinese national standards. They were synthesized by Sangon Biotech (Shanghai, China) Co., Ltd. The sequences are shown in Table 14.

### 4.3. Experimental Methods

#### 4.3.1. DNA Extraction [30]

Take 2 mL of the bacterial suspension collected after culturing in MRS broth or the pre-treated sample lysate, centrifuge at 12,000 rpm for 5 min, discard the supernatant, and collect the bacterial cells. Add 200 μL of lysozyme solution and incubate at 37 °C for 2 h. Then, add 20 μL of proteinase K and 800 μL of CTAB lysis buffer, vortex, and incubate at 56 °C in a water bath overnight. Add an equal volume of Tris-phenol:chloroform/isoamyl alcohol (25:24:1) to the lysis solution, and centrifuge at 13,000 rpm for 10 min. Transfer the supernatant to a new centrifuge tube, add an equal volume of chloroform/isoamyl alcohol (24:1), and centrifuge at 13,000 rpm for 10 min. Transfer the supernatant to a new centrifuge tube, add twice the volume of ice-cold absolute ethanol, mix thoroughly, centrifuge at 13,000 rpm for 5 min, discard the supernatant, add 1 mL of 70% ethanol to the precipitate, wash 2–3 times, air dry naturally, and dissolve in 200 μL of 1× TE buffer. Store at −20 °C.

#### 4.3.2. Determination of DNA Content and Quality

Take 2 μL of the original DNA solution and use a micro-ultraviolet analyzer (DeNovix Inc., Wilmington, DE, USA) to measure the A260, A280, A260/A280, and DNA concentration. A DNA template with an A260/A280 ratio between 1.8 and 2.0 is considered acceptable. Based on the DNA concentration of the original sample solution, select an appropriate dilution for subsequent PCR amplification.

#### 4.3.3. Real-Time Fluorescence PCR Amplification System

##### Experimental Process Control

To ensure the accuracy of the experimental results, set the target strains as the positive control, the non-target strains as the negative control, and ddH_2_O as the blank control during the detection process.

##### Real-Time Fluorescence PCR System

The real-time fluorescence PCR system is 25 μL, including 2 μL of the DNA template, 12.5 μL of PCR Master Mix, and 0.6 μL of each of the forward primer, reverse primer, and probe. Make up the volume with sterilized distilled water (Table 15).

##### Real-Time Fluorescence PCR Parameters

Perform pre-denaturation at 94 °C for 30 s, and then denaturation at 94 °C for 5 s and annealing at 60 °C for 35 s, for 40 cycles. (The reaction parameters can be adjusted appropriately according to the instructions of the selected fluorescence PCR premix and different instruments.)

##### Quality Control

The experiment is considered invalid if any of the following conditions are not met:

(a) Blank control: No fluorescence signal is detected in the fluorescence channel, and the Ct value should be >35.0.
(b) Negative control: No fluorescence signal is detected in the fluorescence channel, and the Ct value should be >35.0.
(c) Positive control: A fluorescence signal is detected in the fluorescence channel, and a typical amplification curve appears, with the Ct value ≤ 30.0.

##### Result Determination

(a) If the Ct value ≤ 30.0, the sample is determined to be positive.
(b) If the Ct value > 35.0, the sample is determined to be negative.
(c) If 30.0 < Ct value ≤ 35.0, repeat the amplification once. If the Ct value is still ≤35.0 after the second amplification, the sample is determined to be positive; if the Ct value > 35.0 after the second amplification, the sample is determined to be negative.

#### 4.3.4. Screening Primer–Probe Combinations for Multiplex Real-Time Fluorescence PCR System

##### Screening of Modified Groups for Multiplex Real-Time Fluorescence PCR

Multiplex real-time fluorescence PCR technology can simultaneously detect multiple target components in one reaction. The key to this technology, in addition to finding the sequence information of specific primers and probes for the target source species, is also to find ideal fluorescence-labeled modification groups for the probes of multiple target components. These fluorescence-labeled modification groups should not have an interaction effect on the emission spectrum, and the fluorescence signals that they generate must be at the same level; otherwise, they cannot be analyzed in the same reaction. Based on this principle, the modification groups for the multiplex real-time fluorescence PCR need to be screened in the early stage of the experiment.

##### Specificity Verification of Multiplex Real-Time Fluorescent PCR Primer–Probe Combination

DNA templates of seven Lactobacillus species, namely *L. rhamnosus, L. plantarum, L. delbrueckii* subsp. *bulgaricus, L. acidophilus, L. casei, L. paracasei*, and *L. reuteri*, were selected. The designed multiplex primer–probes were used to detect the target *Lactobacillus* strains individually to verify the multiple primer–probes’ specificity.

#### 4.3.5. Specificity Verification of Multiplex Fluorescence PCR Primer–Probe

Using the above-validated multiplex primer–probe sets, multiplex real-time PCR amplification was performed on the target bacterial species as well as non-target species in order to verify the specificity of the multiplex primer–probe system.

#### 4.3.6. Detection Limit (Limit of Detection, LOD)

The bacterial suspensions of the target strains were serially diluted and subjected to multiplex real-time fluorescent PCR amplification to explore the detection limit of the established method and verify its sensitivity.

#### 4.3.7. Repeatability Validation of the Multiplex PCR Assay

To verify the accuracy and reproducibility of the established method, mixed bacterial suspensions containing target strains were prepared for repeatability testing. Six independent replicate experiments were performed in parallel using the optimized multiplex real-time fluorescence PCR primer–probe set, in accordance with standardized laboratory protocols, to assess assay repeatability.

#### 4.3.8. Validation Using Commercial Food Samples

Ten commercial food products containing two or more of the target stains were collected and tested using the multiplex primers and probes to verify the feasibility of the method.

## 5. Conclusions

In the food industry, lactic acid bacteria (LAB) have become one of the most preferred functional additives in recent years, with widespread applications in conventional foods, dairy products, and health food products [32]. Among LAB, the genus *Lactobacillus* represents a key functional group involved in fermentation. To achieve diverse functional effects, food manufacturers commonly incorporate two or more distinct *Lactobacillus* species during production.

The FAO/WHO guidelines for the evaluation of probiotics in food clearly emphasize that the beneficial effects of probiotics are strain-specific, and food labels are required to declare the genus, species, and strain designation of the probiotics used, as well as the viable cell count throughout the shelf life [33]. In May 2013, the National Health and Family Planning Commission issued a formal reply to the China Dairy Industry Association (Document No. 367 [2013] of the Office for Food Safety Supervision) [34], which explicitly stipulated that the strain information of Lactobacillus used in prepackaged foods must be clearly labeled. In addition, the Q&A (Revised Edition) of the General Standard for the Labeling of Prepackaged Foods (GB 7718-2011) also provides clear specifications regarding the mandatory labeling of bacterial strains on product packaging [35].

At present, the most widely adopted approach for the identification and detection of LAB in China remains the traditional culture-based microbiological method. This approach is time-consuming and relies primarily on phenotypic characteristics, including colony morphology and physiological–biochemical properties [23,24]. However, variations in culture conditions may lead to inconsistent results for the same strain, and there are notable differences in physiological and biochemical profiles among distinct strains. As a result, the biochemical test results of target strains often cannot fully match the reference criteria provided in standard manuals, hindering accurate and reliable identification. Common molecular biological methods, such as conventional PCR or real-time fluorescence PCR, are also typically dependent on pure single colonies isolated via traditional culture techniques. For complex probiotic products containing multiple *Lactobacillus* species or exhibiting large differences in viable cell concentrations, these methods fail to achieve efficient and accurate simultaneous identification. Furthermore, most current detection techniques focus solely on the identification of a single *Lactobacillus* species, and there remains an urgent shortage of systematic and rapid methods for the simultaneous identification of multiple *Lactobacillus* species.

As of August 2024, the Announcement on Updating the List of Bacterial Strains Permitted for Use in Food Products (No. 4 of 2022) issued by the National Health Commission of the People’s Republic of China [25] authorizes the use of 41 probiotic species in conventional foods, 15 in infant foods, and 21 in health foods. The accurate classification and identification of lactic acid bacteria at the species level represent not only a key research focus but also an essential technical support for food safety supervision in China. Nevertheless, this project focused solely on the study of seven *Lactobacillus* species in this announcement; validated identification methods remain unavailable for other lactic acid bacterial species included in this list. The technical system established in this study lays a critical foundation for the future development of highly specific, sensitive, and high-throughput multiplex real-time fluorescence PCR identification methods for *Lactobacillus* species. The methodology can serve as a reference for the identification of other lactic acid bacterial species. This technical platform provides a reliable basis for government regulatory agencies to supervise the accurate and compliant labeling of *Lactobacillus* species in food products, thereby generating considerable economic and social benefits.

## 6. Patents

Shanghai Institute of Quality Inspection and Technical Research Co., Ltd. (2026). A primer composition for simultaneous detection of four *Lactobacillus* species and its application (No. 202511864320.6). Chinese Invention Patent.

## Figures and Tables

**Table 1:** *L. rhamnosus*, *L. plantarum*, *L. delbrueckii* subsp. *Bulgaricus*, and *L. casei* primer–probe multi-qPCR results.

Primer–Probe Combination	Strain							
	*L. rhamnosus*	*L. plantarum*	*L. delbrueckii* subsp. *bulgaricus*	*L. acidophilus*	*L. casei*	*L. paracasei*	*L. reuteri*	Blank
*L. rhamnosus* (FAM)	21.92	>40	>40	>40	39.92	>40	>40	>40
*L. plantarum* (CY5)	>40	23.62	36.23	>40	38.97	>40	>40	>40
*L. delbrueckii* subsp. *Bulgaricus* (HEX)	>40	>40	21.95	>40	>40	>40	>40	>40
*L. casei* ^1^ (NED)	>40	>40	23.36	>40	20.20	>40	>40	>40

^1^ DNA of *L. delbrueckii* subsp. *bulgaricus* was specifically amplified by the primer–probe set for *L. casei*.

**Table 2:** *L. plantarum*, *L. acidophilus*, *L. delbrueckii* subsp. *Bulgaricus*, and *L. casei* primer–probe multi-qPCR results.

Primer–Probe Combination	Strain							
	*L. rhamnosus*	*L. plantarum*	*L. debrueckii* subsp. *bulgaricus*	*L. acidophilus*	*L. casei*	*L. paracasei*	*L. reuteri*	Blank
*L. plantarum* (CY5)	>40	23.09	36.23	>40	38.97	>40	>40	>40
*L. acidophilus* (ROX)	>40	>40	39.32	23.02	>40	>40	>40	>40
*L. delbrueckii* subsp. *Bulgaricus* (HEX)	>40	>40	21.95	>40	>40	>40	>40	>40
*L. casei* ^1^ (NED)	>40	>40	25.67	>40	19.98	38.65	>40	>40

^1^ DNA of *L. delbrueckii* subsp*. bulgaricus* was specifically amplified by the primer–probe set for *L. casei.*

**Table 3:** *L. rhamnosus*, *L. plantarum*, *L. elbrueckii* subsp. *Bulgaricus*, and *L. paracasei* primer–probe multi-qPCR results.

Primer–Probe Combination	Strain							
	*L. rhamnosus*	*L. plantarum*	*L. debrueckii* subsp. *bulgaricus*	*L. acidophilus*	*L. casei*	*L. paracasei*	*L. reuteri*	Blank
*L. rhamnosus* (FAM)	21.11	>40	>40	>40	38.34	>40	>40	>40
*L. plantarum* (CY5)	>40	22.46	36.75	>40	36.74	>40	>40	>40
*L. delbrueckii* subsp. *Bulgaricus* (HEX)	>40	>40	20.91	>40	>40	>40	>40	>40
*L. paracasei *^1^ (NED)	>40	>40	21.96	>40	19.35	>40	>40	>40

^1^ DNA of *L. delbrueckii* subsp. *bulgaricus* was specifically amplified by the primer–probe set for *L. paracasei*.

**Table 4:** *L. reuteri* and *L. casei* primer–probe multi-qPCR results.

Primer–Probe Combination	Strain							
	*L. rhamnosus*	*L. plantarum*	*L. delbrueckii* subsp. *bulgaricus*	*L. acidophilus*	*L. casei*	*L. paracasei*	*L. reuteri*	Blank
*L. reuteri* (HEX)	>40	>40	>40	>40	>40	>40	20.98	>40
*L. casei* ^1^ (NED)	>40	>40	>40	>40	16.02	>40	21.21	>40

^1^ DNA of *L. reuteri* was specifically amplified by the primer–probe set for *L. casei.*

**Table 5:** *L. reuteri* and *L. paracasei* primer–probe multi-qPCR results.

Primer–Probe Combination	Strain							
	*L. rhamnosus*	*L. plantarum*	*L. debrueckii* subsp. *bulgaricus*	*L. acidophilus*	*L. casei*	*L. paracasei*	*L. reuteri*	Blank
*L. reuteri* (HEX)	>40	>40	>40	>40	>40	>40	21.35	>40
*L. paracasei* ^1^ (NED)	>40	>40	>40	>40	39.25	17.01	21.91	>40

^1^ DNA of *L. reuteri* was specifically amplified by the primers and probe for *L. parcasei.*

**Table 6:** *L. rhamnosus*, *L. plantarum*, *L. acidophilus*, and *L. paracasei* primer–probe multi-qPCR results.

Primer–Probe Combination	Strain							
	*L. rhamnosus*	*L. plantarum*	*L. delbrueckii* subsp. *bulgaricus*	*L. acidophilus*	*L. casei*	*L. paracasei*	*L. reuteri*	Blank
*L. rhamnosus* (FAM)	18.58	37.13	38.1	39.99	38.27	37.98	36.83	>40
*L. plantarum* (CY5)	36.16	16.88	36.4	37.33	36.89	36.28	37.29	>40
*L. acidophilus* (ROX)	38.34	38.14	38.35	17.69	38.40	36.95	36.86	>40
*L. paracasei* (NED)	>40	36.70	>40	38.46	>40	20.55	38.07	>40

**Table 7:** *L. rhamnosus*, *L. plantarum*, *L. acidophilus*, and *L. casei* primer–probe multi-qPCR results.

Primer–Probe Combination	Strain							
	*L. rhamnosus*	*L. plantarum*	*L. delbrueckii* subsp. *bulgaricus*	*L. acidophilus*	*L. casei*	*L. paracasei*	*L. reuteri*	Blank
*L. rhamnosus* (FAM)	21.24	>40	>40	>40	36.97	>40	35.38	>40
*L. plantarum* (CY5)	39.61	17.11	>40	35.06	>40	39.34	37.03	>40
*L. acidophilus* (ROX)	>40	36.11	>40	20.72	39.41	38.88	39.91	>40
*L. casei* (NED)	>40	>40	>40	>40	20.09	>40	>40	>40

**Table 8:** *L. rhamnosus*, *L. plantarum*, *L. delbrueckii* subsp. Bulgaricus, and *L. acidophilus* primer–probe multi-qPCR results.

Primer–Probe Combination	Strain							
	*L. rhamnosus*	*L. plantarum*	*L. delbrueckii* subsp. *bulgaricus*	*L. acidophilus*	*L. casei*	*L. paracasei*	*L. reuteri*	Blank
*L. rhamnosus* (FAM)	19.66	>40	>40	>40	>40	37.48	36.23	>40
*L. plantarum* (CY5)	>40	15.25	>40	>40	38.52	37.69	>40	>40
*L. delbrueckii* subsp. *Bulgaricus* (HEX)	>40	>40	24.26	>40	39.09	>40	>40	>40
*L. acidophilus* (ROX)	>40	>40	>40	16.51	38.24	37.18	>40	>40

**Table 9:** *L. rhamnosus*, *L. plantarum*, *L. acidophilus*, and *L. reuteri* primer–probe multi-qPCR results.

Primer–Probe Combination	Strain							
	*L. rhamnosus*	*L. plantarum*	*L. delbrueckii* subsp. *bulgaricus*	*L. acidophilus*	*L. casei*	*L. paracasei*	*L. reuteri*	Blank
*L. rhamnosus* (FAM)	16.47	38.78	>40	37.23	>40	37.12	37.53	>40
*L. plantarum* (CY5)	38.61	16.39	39.16	38.38	37.31	38.49	37.69	>40
*L. acidophilus* (ROX)	37.22	37.37	38.11	17.05	38.24	36.20	36.18	>40
*L. reuteri* (NED)	>40	>40	>40	>40	>40	39.18	22.88	>40

**Table 10:** Specificity verification of target and non-target strain results.

Strain	Ct1	Ct2	Ct3	Ct4
*L. acidophilus*	18.66	15.15	18.51	18.38
*L. plantarum*	17.02	16.78	17.29	17.36
*L. rhamnosus*	19.55	19.47	16.63	16.54
*L. paracasei*	23.68	22.90	18.77	18.84
*L. delbrueckii* subsp. *bulgaricus*	>40	>40	>40	>40
*L. casei*	>40	>40	>40	>40
*L. reuteri*	>40	>40	>40	>40
*B. animalis*	>40	>40	>40	>40
*S. thermophilus*	>40	>40	>40	>40
*L. lactis*	>40	>40	>40	>40
ddH_2_O	>40	>40	>40	>40

**Table 11:** Limit of detection results.

Target	*L. plantarum*			*L. rhamnosus*			*L. paracasei*			*L. acidophilus*		
Concentration of standard strain bacterial suspension	Plate Count	Ct Value	Results	Plate Count	Ct Value	Results	Plate Count	Ct Result	Results	Plate Count	Ct Value	Results
	CFU/g			CFU/g			CFU/g			CFU/g		
	1.3 × 10^8^	16.85	Pos.	3.6 × 10^7^	14.24	Pos.	1.1 × 10^8^	19.81	Pos.	1.4 × 10^6^	17.49	Pos.
	1.3 × 10^7^	21.92	Pos.	3.6 × 10^6^	18.54	Pos.	1.1 × 10^7^	23.47	Pos.	1.4 × 10^5^	20.82	Pos.
	1.3 × 10^6^	29.01	Pos.	3.6 × 10^5^	24.91	Pos.	1.1 × 10^6^	28.05	Pos.	1.4 × 10^4^	25.54	Pos.
	1.3 × 10^5^	31.48	Pos.	3.6 × 10^4^	28.39	Pos.	1.1 × 10^5^	32.04	Pos.	1.4 × 10^3^	28.67	Pos.
	1.3 × 10^4^	34.22	Pos.	3.6 × 10^3^	31.23	Pos.	1.1 × 10^4^	39.48	Neg.	1.4 × 10^2^	32.06	Pos.
	1.3 × 10^3^	38.16	Neg.	3.6 × 10^2^	35.36	Neg.	1.1 × 10^3^	39.37	Neg.	1.4 × 10^1^	35.41	Neg.
	1.3 × 10^2^	39.06	Neg.	3.6 × 10^1^	>40	Neg.	1.1 × 10^2^	39.47	Neg.		36.35	Neg.
Positive target strain	/	23.33	Pos.	/	19.22	Pos.	/	26.89	Pos.	/	22.75	Pos.

**Table 12:** Repeatability of measurement results.

Sample	No.	Primer–Probe Combination	Ct Value	Result	RSD%
Mixed bacterial suspensions of *L. acidophilus*, *L. plantarum*, *L. rhamnosus*, *and L. paracasei*	1	*L. acidophilus*	16.60	17.02 ± 0.35	2.07%
		*L. plantarum*	16.63	17.02 ± 0.77	4.36%
		*L. rhamnosus*	19.23	20.08 ± 1.10	5.48%
		*L. paracasei*	20.78	21.14 ± 0.47	2.21%
	2	*L. acidophilus*	17.19		
		*L. plantarum*	17.24		
		*L. rhamnosus*	19.08		
		*L. paracasei*	20.54		
	3	*L. acidophilus*	16.75		
		*L. plantarum*	17.21		
		*L. rhamnosus*	19.21		
		*L. paracasei*	20.98		
	4	*L. acidophilus*	17.25		
		*L. plantarum*	18.52		
		*L. rhamnosus*	20.12		
		*L. paracasei*	21.79		
	5	*L. acidophilus*	17.52		
		*L. plantarum*	17.56		
		*L. rhamnosus*	21.23		
		*L. paracasei*	21.25		
	6	*L. acidophilus*	16.82		
		*L. plantarum*	18.54		
		*L. rhamnosus*	21.58		
		*L. paracasei*	21.52		

**Table 13:** Commercial food sample results.

No.	Sample	Components	Primer–Probe Combination	Ct Value	Result
1	Health food product 1	*L. plantarum* Wk86, *L. acidophilus* LA16, *L. plantarum* Lp99, *L. casei* LC89, *L. plantarum* N13, *B. longum* BL44, *B. bifidum* BBi77, B. breve BBr02, *L. paracasei* LC28, *L. rhamnosus* BA12, *L. delbrueckii* subsp. *bulgaricus* LB50, *B. adolecentis* BAC30, *Bacilus coagulans* BC99	*L. acidophilus*	18.55, 18.91, 22.99	Detected
			*L. plantarum*	16.34, 16.19, 16.51	Detected
			*L. rhamnosus*	19.63, 20.51, 20.13	Detected
			*L. paracasei*	22.58, 24.30, 22.99	Detected
2	Health food product 2	*L. acidophilus*; *B. longum*; *L. plantarum*	*L. acidophilus*	21.56, 22.71, 21.56	Detected
			*L. plantarum*	21.54, 20.51, 19.61	Detected
			*L. rhamnosus*	>40, >40, >40	Not Detected
			*L. paracasei*	>40, >40, >40	Not Detected
3	Health food product 3	*L. acidophilus*, *Bifidobacterium*, *L. paracasei*	*L. acidophilus*	22.21, 19.92, 20.35	Detected
			*L. plantarum*	>40, >40, >40	Not Detected
			*L. rhamnosus*	>40, >40, >40	Not Detected
			*L. paracasei*	30.61, 30.23, 30.21	Detected
4	Health food product 4	*L. fermentum*, *L. acidophilus*, *L. paracasei*	*L. acidophilus*	20.73, 22.05, 22.25	Detected
			*L. plantarum*	>40, >40, >40	Not Detected
			*L. rhamnosus*	>40, >40, >40	Not Detected
			*L. paracasei*	22.82, 28.98, 28.58	Detected
5	Health food product 5	*L. plantarum*, *L. acidophilus*, *B. longum*	*L. acidophilus*	24.25, 26.07, 24.04	Detected
			*L. plantarum*	26.44, 29.14, 27.35	Detected
			*L. rhamnosus*	>40, >40, >40	Not Detected
			*L. paracasei*	>40, >40, >40	Not Detected
6	Solid beverage 1	*L. acidophilus* NCFM, *B. lactis* Bi-07, *B. animalis* Bb-12, *L. rhamnosus* LGG, *L. rhamnosus* HN001, *B. lactis* HN019	*L. acidophilus*	24.95, 24.11, 23.63	Detected
			*L. plantarum*	>40, >40, >40	Not Detected
			*L. rhamnosus*	24.98, 24.36, 23.99	Detected
			*L. paracasei*	>40, >40, >40	Not Detected
7	Solid beverage 2	*L. plantarum*, *L. rhamnosus*, *P. acidilactici*, *B. lactis*; lactic acid bacteria compound powder (*L. helveticus* R0052, *L. plantarum* R1012, *B. longum* R0175, *L. paracasei* Lpc-37)	*L. acidophilus*	>40, >40, >40	Not Detected
			*L. plantarum*	18.91, 18.81, 19.43	Detected
			*L. rhamnosus*	24.15, 24.29, 25.22	Detected
			*L. paracasei*	32.17, 31.88, 32.62	Detected
8	Solid beverage 3	*L. plantarum*, *L. rhamnosus*, *B. lactis*; probiotic powder (*L. plantarum* TSP05, *L. reuteri* TSR332, *L. fermentum* TSR331, *B. lactis* CP-9, *B. infantis*, *L. rhamnosus* bv-77); lactic acid bacteria compound powder (*L. plantarum* HA119, *L. casei* R0215, *L. helveticus* R0052); *L. acidophilus* NCFM, *B. lactis* CECT8154, *L. rhamnosus* CNCMI-4036, *B. breve* B-3, *B. lactis* HN019, *L. rhamnosus* HN001, *B. lactis* Bi-07, *B. breve* M-16V, *B. longum* BB536	*L. acidophilus*	30.58, 28.35, 30.25	Detected
			*L. plantarum*	21.62, 20.11, 21.25	Detected
			*L. rhamnosus*	23.89, 22.15, 23.63	Detected
			*L. paracasei*	>40, >40, >40	Not Detected
9	Solid beverage 4	*B. lactis* HN019, *B. lactis* Bi-04, *L. paracasei* Lpc-37, *L. acidophilus* NCFM	*L. acidophilus*	20.10, 22.51, 22.25	Detected
			*L. plantarum*	38.86, 38.33, 38.86	Not Detected
			*L. rhamnosus*	>40, >40, >40	Not Detected
			*L. paracasei*	29.32, 28.76, 28.52	Detected
10	Solid beverage 5	*L. plantarum*, *B. lactis*, *L. casei*, *L. rhamnosus*, *B. breve*, *L. acidophilus*, *L. paracasei*	*L. acidophilus*	23.09, 22.26, 23.15	Detected
			*L. plantarum*	14.46, 14.17, 14.25	Detected
			*L. rhamnosus*	27.78, 27.58, 27.31	Detected
			*L. paracasei*	32.17, 25.34, 26.32	Detected

**Table 14:** Sequences of primers and probes.

Target	Primer and Probe Sequences
*L. rhamnosus* [24]	F :5′-GGTTGATTCAGTGGCAGCTC-3′ R :5′-GTGTGCATCACCCATGTCC-3′ P :5′-TCAATTTCTGCGCGCGGTACCA-3′
*L. plantarum* [24]	F :5′-AGCTTGAAAGATGGCTTCGG-3′ R :5′-GGTCGGCTACGTATCATTGC-3′ P :5′-ACGCCGCGGGACCATCCAAA-3′
*L. reuteri* [24]	F :5′-CTTTCGCAGCCTGATAGTGG-3′ R :5′-TCCGAAGAGCCTGAGACATC-3′ P :5′-CGGTTGCAGCATTAGTTCCTGC-3′
*L. acidophilus* [24]	F :5′-GAGCTGAACCAACAGATTCAC-3′ R :5′-GCAGGTTCCCCACGTGTTAC-3′ P :5′ -CCCATCCGCCGCTAGCGTT-3′
*L. delbrueckii* subsp. *bulgaricus* [24]	F :5′-ACTTTAGCCCATACCTGCGT-3′ R :5′-GTAAATTCCAAGCCGCCCTT-3′ P :5′-CCGGTTGCCCGTTTCCTGCGG-3′
*L. casei* [24]	F :5′-GCCGGGATCTTCAACTCAAC-3′ R :5′-GGACGGCGCAGAAATCTATC-3′ P :5′-TCGCCCAATGCAGCCTGCGC-3′
*L. paracasei* [31]	F :5′-CCG(T)GGTGCATTGGTGATT-3′ R :5′-CACATCCCCGCCTTTGATC-3′ P :5′-CGCCCCCGTCAGCAATGTTGTC-3′

**Table 15:** Final concentration of each reagent in the PCR System.

Reagent	Concentration	Volume/μL
PCR Master Mix (2×)	1×	12.5 μL
Forward primer (5 μmol/L)	200 nmol/L	0.6 μL
Reverse primer (5 μmol/L)	200 nmol/L	0.6 μL
Probe (5 μmol/L)	200 nmol/L	0.6 μL
DNA template (5–50 ng/μL)	/	2 μL
ddH_2_O	/	Make up to 25 μL

## Data Availability

The data presented in this study are available from the corresponding author upon reasonable request.
